# Wireless Connection between Guide Wires and Bone Cement: Extravasated Methyl Methacrylate Mimicking a Retained Guide Wire

**DOI:** 10.1155/2013/180735

**Published:** 2013-05-13

**Authors:** Kevin C. Ching, Avinash Medsinge, Vikas Agarwal, Robert F. Short, Nikhil B. Amesur

**Affiliations:** Department of Radiology, University of Pittsburgh Medical Center, 200 Lothrop Street, 3950 Presby South Tower, Pittsburgh, PA 15213, USA

## Abstract

We present the case of a 56-year-old double lung transplant recipient with chest pain who underwent an attempted endovascular retrieval of what was described as a retained guide wire in the azygos vein. After successfully grasping the tip, the object further migrated to the right pulmonary artery complicating the retrieval. It was realized that the “wire” was extravasated methyl methacrylate from a recent percutaneous kyphoplasty. This is believed to be the first report of attempted endovascular retrieval of extravasated methyl methacrylate in the azygos system. We include the details of this case and briefly review the current literature on the management of extravasated methyl methacrylate from vertebral augmentation procedures. Extravasated methyl methacrylate in the venous system is a common finding after vertebral augmentation procedures and any radiopaque stripe arising from a cemented vertebral body should be first described as probable cement leakage.

## 1. Introduction

Percutaneous vertebroplasty and kyphoplasty are increasingly utilized for rapid pain relief and increased mobilization in patients with vertebral body compression fractures. While major complications are rare, extravertebral leakage of methyl methacrylate has been reported to occur in up to 73% of vertebral augmentation procedures with venous leaks occurring in up to 24% of cases [1]. The vast majority of patients with cement leakage and even cement pulmonary embolism remain asymptomatic. Occasionally in symptomatic patients, embolized intravascular cement has been retrieved using endovascular snares. Endovascular foreign body retrieval is not only a beneficial procedure for patients, but also for physicians as most retained objects are iatrogenic in origin [2]. We describe a patient who presented to the emergency department with chest pain and what was believed to be a retained guide wire in the azygos vein found on chest imaging. Attempted snare retrieval was performed, and after further migration, the foreign body was discovered to be extravasated methyl methacrylate from prior vertebral body augmentation. 

## 2. Case Report

**Figure 1:**
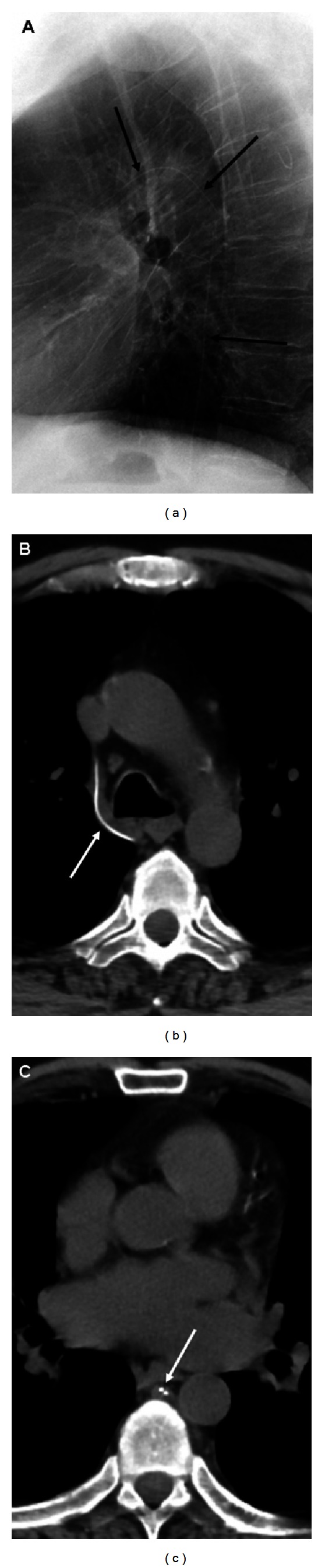
Lateral chest radiograph (a) and unenhanced axial chest CT images (b-c) show an opaque curvilinear density *arrows* within the azygos vein measuring approximately 29 cm in length. A short overlap *arrow *is present between the proximal and distal fragments (c).

**Figure 2:**
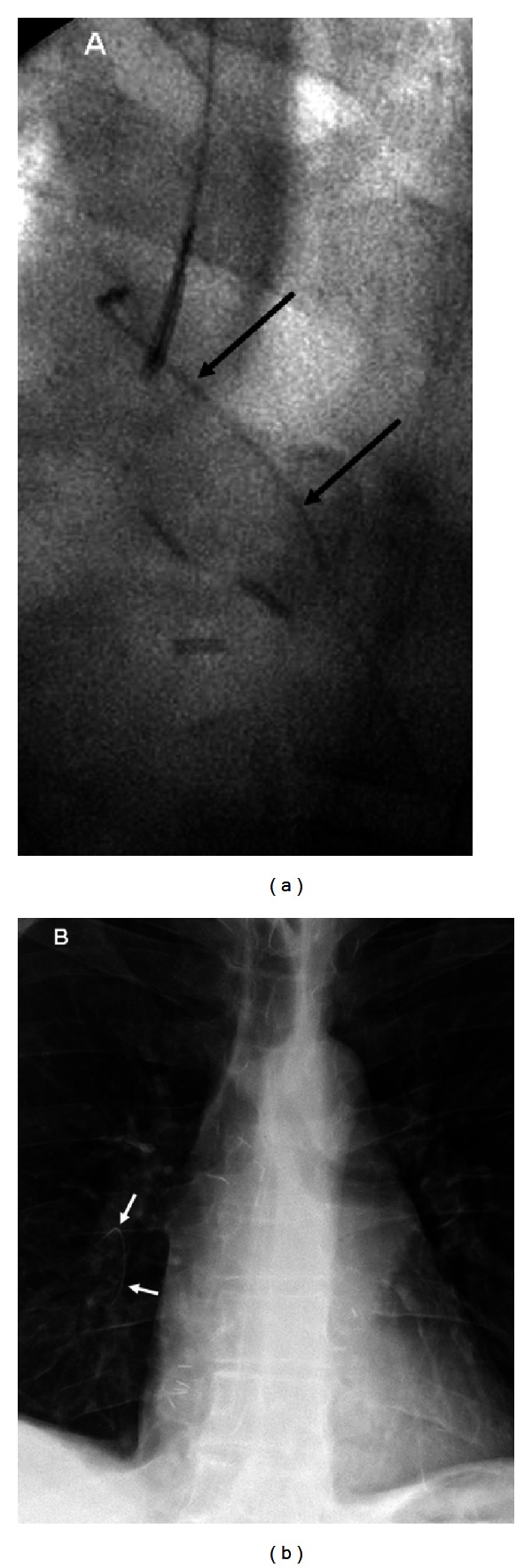
Fluoroscopic image (a) shows the foreign body successfully grasped with a 5-French goose neck snare. Chest radiograph (b) shows the embolized proximal fragment *arrows *within the right lower lobe pulmonary artery.

**Figure 3:**
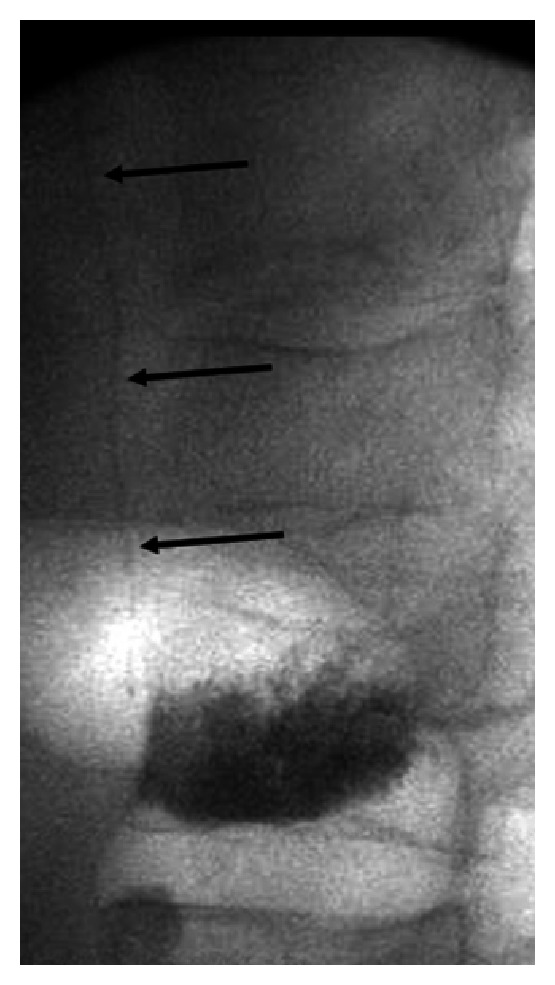
Fluoroscopic image from the T12 kyphoplasty immediately following cement injection shows the vertically oriented methyl methacrylate *arrows* within the azygos vein.

A 56-year-old male with history of double lung transplantation for emphysema presented to the emergency department with chest pain. Initial PA and lateral chest radiographs showed a curvilinear density in the azygos vein (Figure 1(a)) which was thought to be a retained guide wire, likely from one of multiple previous PICC placements. A chest CT confirmed the “wire” within the azygos vein beginning at the T12 level and extending superiorly with the distal tip projecting into the superior vena cava with a total length of approximately 29 cm (Figures 1(b)-1(c)). The “wire” was noted to be fragmented with a small overlap of the segments at the T6 level. The patient was admitted for further evaluation of his chest pain, and interventional radiology was consulted the next day for endovascular foreign body retrieval. With the patient in the supine position, the right internal jugular vein was accessed, and a 6-French sheath was placed. Using a 5-French Amplatz gooseneck snare, the tip of the object was successfully grasped (Figure 2(a)), however, it was difficult to remove and appeared to stretch during the snaring process. During the extraction, the superior fragment migrated to the right lower lobe pulmonary artery leaving the inferior portion in place distally. The right pulmonary artery was then selectively catheterized, and attempts were made to snare the now embolized fragment. After multiple tries using various catheters, retrieval of the fragment within the right pulmonary artery was unsuccessful (Figure 2(b)). It was decided to leave the remaining inferior fragment within the azygos vein, and the procedure was terminated. Soon after the procedure, further inquiry into the patient's history revealed that the patient had recently undergone a percutaneous kyphoplasty for an acute T12 vertebral compression fracture and that the “wire” actually represented venous extravasation of methyl methacrylate (Figure 3). After an extensive inpatient work up, the exact cause of the patient's chest pain was not determined; however, the etiology was believed to be unlikely from the extravasated methyl methacrylate as his chest pain symptoms abated and have not recurred. Prophylactic anticoagulation was thought to be unnecessary, and the patient was discharged. A four-month follow-up, CT showed the methyl methacrylate fragments unchanged in position within the right lower lobe pulmonary artery and azygos vein without complicating imaging features. The patient has had no further issues related to either the vertebral augmentation or attempted endovascular retrieval procedure. 

## 3. Discussion

Vertebroplasty and kyphoplasty are recognized to help alleviate pain from acute vertebral body compression fractures. While vertebral augmentation is safe, venous extravasation of methyl methacrylate is known to occur in up to 24% of procedures with the anterior external venous plexus being the most common location followed by the azygos vein [1, 3]. The vast majority of these patients remains asymptomatic and without complications from the extraosseous cement. Kyphoplasty was developed in an attempt to reduce the rate of cement leakage and restore anatomic osseous configuration. Inflation of a balloon tamp to create a space within the vertebral body allows for lower pressure injection of cement in comparison to the relatively higher pressure injection in vertebroplasty. Reduced rates of extravertebral leakage with kyphoplasty in comparison to vertebroplasty have been shown with contrast injection [4]. 

Extravasated venous methyl methacrylate that travels to the lungs results in cement pulmonary embolism. This type of embolization has been reported to occur in 4.6%–26% of vertebral augmentations depending on whether radiograph or CT was used for postprocedural imaging [1, 3]. The only statistically significant risk factor for cement pulmonary embolism is venous extravasation into the azygos vein or IVC [3, 5]. 

Management of cement pulmonary embolism is somewhat controversial. Most agree that peripheral cement pulmonary emboli in asymptomatic patients should be managed conservatively without instituting anticoagulation therapy [1, 5, 6]. When central cement emboli occurred or the patient was clearly symptomatic, large segments of methyl methacrylate have been removed from the central veins and pulmonary arteries using endovascular snares and even open heart surgery [7–9]. Had the extravasated azygos methyl methacrylate in our patient been correctly identified on the initial radiographic and CT imaging, retrieval efforts would not have been immediately pursued, as it was an unlikely source of our patient's complaint of chest pain. Risk of thrombosis or damage to the azygos vein as a result of leaving the foreign body in place is minimal as long-term venous catheters have been placed in the azygos system with good results in patients with severe central venous stenosis [10].

In a report of intravascular retained surgical items, 8 out of 8 retained guide wires included in the study were located within the nonazygos central circulation [2]. Presentation of the retained intravascular items was often delayed as 7 out of 13 total retained items were missed on immediate postprocedure imaging with some foreign bodies discovered up to 6 weeks later. Patients tend to present with symptoms that while unlikely to be caused by the retained item itself, necessitate further imaging leading to the discovery of the foreign body. 

 Due to this patient's complicated history of lung transplantation and subsequent hospitalizations requiring multiple peripherally inserted central catheters, the curvilinear opaque foreign body was described as a retained guide wire by multiple subspecialty radiology attendings and fellows who reviewed the studies. While no serious morbidity occurred as a result of the attempted snare retrieval and subsequent cement embolization, it is clear that azygos methyl methacrylate can mimic the appearance of a retained guide wire. Because extravasated methyl methacrylate in the venous system is a common finding after vertebral augmentation procedures, any radiopaque stripe arising from a cemented vertebral body should be first described as probable cement leakage.
